# Fluorinated hydroxyapatite conditions a favorable osteo-immune microenvironment via triggering metabolic shift from glycolysis to oxidative phosphorylation

**DOI:** 10.1186/s12967-024-05261-0

**Published:** 2024-05-08

**Authors:** Kaidi Chen, Seongmin Ha, Leyao Xu, Chengwu Liu, Yuanxiang Liu, Xiayi Wu, Zhipeng Li, Shiyu Wu, Bo Yang, Zhuofan Chen

**Affiliations:** 1 Hospital of Stomatology, Sun Yat-sen University, Guangzhou, China; 2 Guangdong Provincial Key Laboratory of Stomatology, Guangzhou, China; 3 Guanghua School of Stomatology, Sun Yat-sen University, Guangzhou, China

**Keywords:** Metabolic shift, Osteo-immunomodulation, Bone regeneration, Fluoride, Macrophages

## Abstract

**Background:**

Biological-derived hydroxyapatite is widely used as a bone substitute for addressing bone defects, but its limited osteoconductive properties necessitate further improvement. The osteo-immunomodulatory properties hold crucial promise in maintaining bone homeostasis, and precise modulation of macrophage polarization is essential in this process. Metabolism serves as a guiding force for immunity, and fluoride modification represents a promising strategy for modulating the osteoimmunological environment by regulating immunometabolism. In this context, we synthesized fluorinated porcine hydroxyapatite (FPHA), and has demonstrated its enhanced biological properties and osteogenic capacity. However, it remains unknown whether and how FPHA affects the immune microenvironment of the bone defects.

**Methods:**

FPHA was synthesized and its composition and structural properties were confirmed. Macrophages were cultured with FPHA extract to investigate the effects of FPHA on their polarization and the related osteo-immune microenvironment. Furthermore, total RNA of these macrophages was extracted, and RNA-seq analysis was performed to explore the underlying mechanisms associated with the observed changes in macrophages. The metabolic states were evaluated with a Seahorse analyzer. Additionally, immunohistochemical staining was performed to evaluate the macrophages response after implantation of the novel bone substitutes in critical size calvarial defects in SD rats.

**Results:**

The incorporation of fluoride ions in FPHA was validated. FPHA promoted macrophage proliferation and enhanced the expression of M2 markers while suppressing the expression of M1 markers. Additionally, FPHA inhibited the expression of inflammatory factors and upregulated the expression of osteogenic factors, thereby enhancing the osteogenic differentiation capacity of the rBMSCs. RNA-seq analysis suggested that the polarization-regulating function of FPHA may be related to changes in cellular metabolism. Further experiments confirmed that FPHA enhanced mitochondrial function and promoted the metabolic shift of macrophages from glycolysis to oxidative phosphorylation. Moreover, in vivo experiments validated the above results in the calvarial defect model in SD rats.

**Conclusion:**

In summary, our study reveals that FPHA induces a metabolic shift in macrophages from glycolysis to oxidative phosphorylation. This shift leads to an increased tendency toward M2 polarization in macrophages, consequently creating a favorable osteo-immune microenvironment. These findings provide valuable insights into the impact of incorporating an appropriate concentration of fluoride on immunometabolism and macrophage mitochondrial function, which have important implications for the development of fluoride-modified immunometabolism-based bone regenerative biomaterials and the clinical application of FPHA or other fluoride-containing materials.

**Graphical Abstract. FPHA was successfully prepared through the chemical and thermal process. The immunomodulatory effects of FPHA were investigated through in vitro and in vivo studies, revealing its ability to induce a metabolic shift in macrophages from glycolysis to mitochondrial oxidative phosphorylation (OxPhos). This metabolic remodeling resulted in a notable suppression of M1 macrophage polarization and promotion of M2 macrophage polarization. Furthermore, FPHA was found to enhance osteogenic differentiation and facilitate bone repair. These findings underscore the promising potential of FPHA as a biomaterial for bone regenerative applications, providing valuable insights for the development of bioactive materials with metabolic-immunoregulatory properties:**

**Graphical Abstract:**

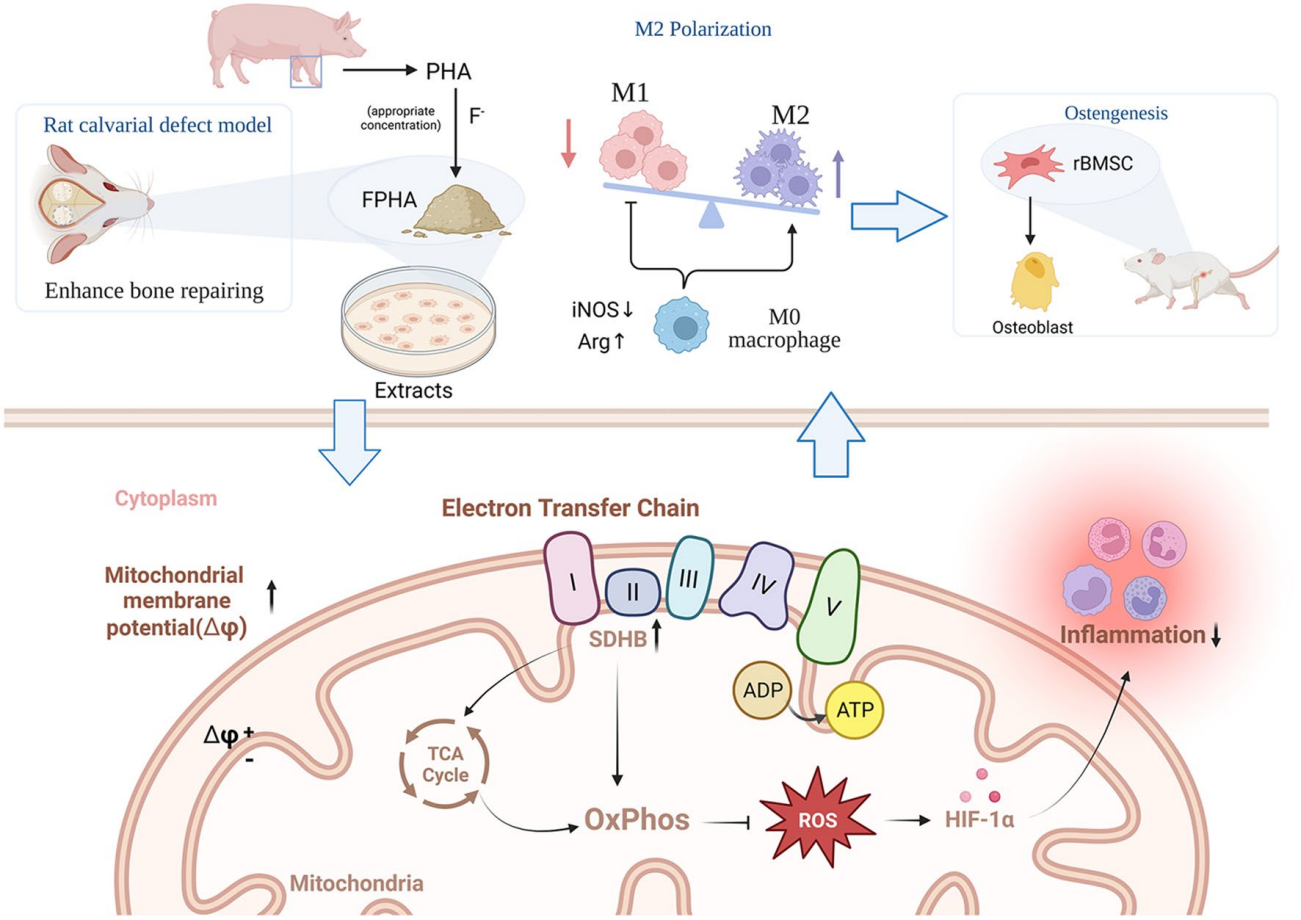

**Supplementary Information:**

The online version contains supplementary material available at 10.1186/s12967-024-05261-0.

## Background

Bone defects, a serious clinical problem caused by tumors, trauma, inflammation, etc., trigger the development of smart biomaterials as bone substitutes for regeneration [1, 2]. The osteo-immunomodulatory properties of biomaterials have emerged as crucial factors in maintaining bone homeostasis and have garnered significant attention [3]. Among immune cells, macrophages play a vital role [3, 4], and the functional polarization of macrophages is pivotal in determining the quality and efficacy of injury repair [5]. Macrophage polarization involves two main states: M1 macrophages promote an inflammatory response, while M2 macrophages possess the ability to suppress inflammation and regulate tissue repair and regeneration [5, 6]. Following the implantation of biomaterials, the incipient acute inflammation was triggered, leading to increased release of cytokines and reactive oxygen species (ROS). The elevated inflammation levels and excessive ROS can result in phagocytose and degrade the implanted biomaterials [7], potentially leading to broader detrimental effects [8–10]. Hence, precise regulation of macrophage activation is essential for tissue homeostasis maintenance and effectively reducing the ROS and inflammatory impact caused by material implantation. In this context, immunometabolism has been a subject of extensive research in recent years, as different macrophage activation states exhibit distinct metabolic profiles [5, 11]. M1 polarization is characterized by increased glycolysis and pentose phosphate pathway (PPP) activity, coupled with reduced oxidative phosphorylation (OxPhos), and in contrast, M2 macrophages display effective OxPhos and reduced PPP activity [6, 12]. Meanwhile, glycolysis and OxPhos uniquely regulate macrophage phenotype and function [13–15]. Therefore, manipulating factors that influence macrophage metabolism could potentially be utilized to modulate macrophage polarization [16].

Fluoride plays a crucial role in the regulation of bone tissue regeneration, repair, and remodeling [14, 17]. Notably, fluoride exerts its effects in a concentration-dependent manner within the human body [18], which shows diverse effects on the metabolism and function of immune cells [14, 19, 20]. Appropriate concentration of fluoride ions was demonstrated to enhanced M2 macrophage polarization [21], via a potential metabolic pathway including the mitochondrial tricarboxylic acid (TCA) cycle and OxPhos [14, 22]. Taken together, fluoride modification may be a promising strategy for modulating the osteoimmunology environment via regulating immunometabolism.

In our previous studies, FPHA was successfully synthesized using a chemical and thermal method [23]. FPHA displayed superior physicochemical and biological properties compared to PHA, particularly when prepared from a 0.25 M NaF aqueous solution [24]. Additionally, fluoride incorporation enhances the osteogenic activity of both BMSCs and MG63 cells [21, 25, 26]. In vivo experiments [26, 27] demonstrated that fluoride incorporation exhibited a greater osteogenic ability. Moreover, clinical trials have been conducted to evaluate the efficacy and safety of FPHA. However, the underlying mechanism related to fluoride regulating immunometabolism remains unveiled.

Therefore, the objective of this study was to investigate the impact of FPHA on macrophage polarization and the underlying mechanism related to immunometabolism. In this research, we proved that FPHA can actively enhance macrophage mitochondrial function and promote a metabolic shift from glycolysis to mitochondrial oxidative phosphorylation. Consequently, FPHA induced M2 polarization and suppressed M1 polarization, creating a favorable osteo-immune microenvironment. These findings may provide valuable insights into the impact of incorporating an appropriate concentration of fluoride on immunometabolism and macrophage mitochondrial function, which have important implications for development of immunometabolism-based fluoride-containing biomaterials.

## Methods

### FPHA preparation, characterization and extract preparation

PHA and FPHA were synthesized using a chemical and thermal process previously established by our research group [25–27]. The surface morphology of PHA and FPHA was observed using scanning electron microscope (SEM, MIRA LM, Tescan, Czech Republic). Additionally, elemental characterization and distribution analysis were conducted using energy-dispersive X-ray spectroscopy (EDS) mode. Fourier-transform infrared spectroscopy (FTIR, Nicolet NXR 9650, Thermo-Fisher, USA) were utilized to identify crystal phases and specific chemical groups present in PHA and FPHA. The changes in crystallinity of PHA and FPHA were investigated using X-ray diffraction (XRD, Empyrean, Malvern Panalytical, Netherlands).

Extract were prepared following the ISO/EN 10993-5 standard, as previously described in another study [26]. The sterilized samples were then placed into serum-free DMEM (Gibco, USA) in a ratio of 100 mg to 1 ml. To ensure a more uniform interaction between the DMEM and the materials, the centrifuge tubes were positioned horizontally and incubated at 37 °C for 24 h. Subsequently, the supernatant was collected following centrifugation and filtrated through a 0.22 μm filter membrane (Merck Millipore, USA). The concentration of F ions in the solution was determined using a fluorine selective electrode (PF-202-C; Leici, China) connected to an ion analyzer (Origin Dual Star; Thermo Scientific, USA). For subsequent experiments, the PHA and FPHA extract was supplemented with 10% fetal bovine serum (FBS, A115-500, Nobimpex, Germany) and 1% penicillin/streptomycin.

### Cell proliferation and viability

The biocompatibility of FPHA was evaluated by examining the proliferation and viability of RAW264.7 macrophages. The cells were seeded in 24-well plates at a density of 1 × 10^5^ cells per well. Subsequently, they were cultured with DMEM, PHA extract, or FPHA extract for 1, 2, and 3 days. The proliferation of macrophages was assessed using the cell counting kit-8 (CCK-8, Dojindo Laboratories, Japan) assay, while the viability of macrophages was evaluated using the Calcein-AM/PI Live-Dead Cell Staining Kit (Solarbio, China).

### In vitro macrophage responses following culture with PHA and FPHA extract

#### Cell culture and macrophage activation

RAW264.7 macrophages (TCM-13) were obtained from the Cell Bank at the Shanghai Institute of Biochemistry and Cell Biology, China. The cells were cultured in DMEM supplemented with 10% FBS and 1% penicillin/streptomycin (Gibco, USA), and maintained in a 5% CO_2_ humidified atmosphere at 37 °C. Before using the FBS, it was inactivated by heating at 56 °C for 30 min. When the cells reached approximately 80% confluence, they were passaged by scraping.

To investigate the modulatory effects of FPHA on macrophages, LPS (L2630, Merck Millipore, USA) was used to activate the macrophages. The culture medium was replaced with medium containing 1000 ng/mL LPS and the cells were stimulated for 6 h. A previous study [28] demonstrated that macrophages exhibit rapid responsiveness to LPS stimulation, with the 6-hour time point being optimal. Stimulation period shorter than 6 h may fail to distinguish the intensity of different stimulators, while stimulation time longer than 6 h may lead to misjudgment of some highly potent stimulators [28]. Afterwards, the LPS-activated RAW264.7 macrophages were cultured with 1 ml DMEM, PHA extract, and FPHA extract in 6-well plates for subsequent experiments. Cells cultured with DMEM were served as a control. Building upon the findings of the previous study [28] and pre-experimental results, the peak hour of pro-inflammatory gene expression was 6 h. And considering the delayed protein expression, 24 h is a better time point for intracellular protein assessment, while 48 h may be a more suitable time for evaluating secretory protein levels.

#### qRT-PCR

To investigate the effects of the extract on macrophages at the gene level, qRT-PCR was performed. After culturing the macrophages with the extract for 6 h and 1 day, total RNA was isolated using the RNA Purification Kit (Esunbio, China). The quality and quantity of the total RNA were assessed using a spectrophotometer (Nanodrop One, Thermo Fisher Scientific, USA). Then, cDNA synthesis was performed using a reverse transcription kit (Hifair® III 1st Strand cDNA Synthesis SuperMix, Yeasen, China) with 500ng of RNA as the starting material, following the manufacturer’s instructions. The qRT-PCR analysis was conducted using the qPCR SYBR Mix (Hieff® qPCR SYBR Green Master Mix No Rox, Yeasen, China) with a 10 µl reaction volume, employing the LightCycler® 96 instrument (Roche, Switzerland). The primer sequences used for qRT-PCR are listed in Table S1.

#### Western blot

To investigate the effects of the extract on macrophages at the protein level, Western blot analysis was performed. After culturing the macrophages with the extract for 1 day, the cells were lysed using RIPA lysis buffer. The protein concentration in the lysate was determined using the BCA Protein Assay Kit (CW0014, Cwbio, China). Subsequently, equal amounts of protein from each sample were loaded onto SDS-PAGE gels (SurePAGE™, GenScript, China) and separated by electrophoresis. The separated proteins were then transferred to a PVDF membrane (Merck Millipore, USA). To block non-specific binding sites, the membrane was incubated in 5% w/v skim milk (1172, Biofroxx, Germany) for 90 min. Next, the membrane was incubated overnight at 4 °C with primary antibodies diluted in appropriate concentrations. The primary antibodies used in this study included anti-iNOS antibody (1:1000, ab178945, Abcam, UK), anti-Arg antibody (1:100, 93668T, CST, USA), anti-IL10 antibody (1:500, Affinity, China), anti-MMP9 antibody (1:1000, Proteintech, USA) and anti-β-tubulin antibody (1:2500, #2128S, CST, USA). After washing, the membrane was incubated with a Goat anti-Rabbit secondary antibody (1:8000; #7074s, CST, USA). The protein bands were visualized using the Immobilon Western HRP Substrate (WBKLS0100, Merck Millipore, USA) and captured using a ChemiDoc XRS System (BioRad, Hercules, USA).

#### Enzyme linked immunosorbent assay (ELISA) of cytokines

To determine the levels of TNFα and TGFβ1, two secreted proteins, in the culture media supernatant after a 48-hour incubation, ELISA kits were utilized. The ELISA kits used in this study were for TNFα (Cusabio, China) and TGFβ1 (Mlbio, China). The protocols provided by the manufacturers were followed to measure the concentrations of TNFα and TGFβ1 in the supernatant samples. It is worth mentioning that prior to TGFβ1 protein detection, the test samples should be activated with HCl and NaOH according to the instructions, which facilitates the release and subsequent detection of TGFβ1 in the samples.

#### Nitric oxide (NO) release detection

After culturing the cells for 1 day, the production of nitrate and nitrite anions in the culture media supernatant was assessed using the Griess method. This was done using the Total Nitric Oxide Assay Kit (Beyotime Biotechnology, S0023) according to the manufacture’s protocol.

### In vitro effects of macrophage-conditioned medium on osteogenesis

#### Isolation and culture of rat bone marrow-derived mesenchymal stem cells (rBMSCs)

The rBMSCs were isolated from the bone marrow of three-week-old male Sprague-Dawley rats, following a previously described protocol [26]. Briefly, the bone marrow cells were obtained by flushing the tibias and femurs with complete medium with minimum essential medium alpha (α-MEM, Gibco, USA) supplemented with 10% FBS and 1% penicillin-streptomycin. The collected bone marrow cells were centrifuged at 1200 rpm for 5 min and then resuspended in 10 mL of complete medium. The suspended cells were seeded into 75 cm^2^ culture flasks (Corning, USA). The cells were cultured in a humidified atmosphere with 5% CO_2_ at 37 °C. After 48 h, the adherent cells, referred to as passage 0, were collected and further cultured. For subsequent experiments, the cells were passaged using TrypLE™ Express (Gibco, USA) when they reached approximately 80% confluence. The passage 3 to 5 cells were used in the subsequent experiments.

#### Macrophage-conditioned medium preparation

Macrophages were cultured with three types of culture media: DMEM, PHA extract, and FPHA extract. After 1 day of incubation, the culture supernatants were extracted and collected. The collected supernatants were subjected to centrifugation at 1500 rpm for 10 min at 4 °C and stored at -80 °C for subsequent experiments.

#### Osteogenic differentiation

The rBMSCs (1.0 × 10^5^ cells per well) were cultured in 24-well plate with co-stimulation medium consisting of osteogenic induction medium and macrophage-conditioned medium with different treatments at a ratio of 1:1. The rBMSCs cultured for 7 days were stained with Alkaline Phosphatase Color Development Kit (C3206, Beyotime, China). Alizarin red S (ARS, Pricella, China) staining was performed on day 14 to detect mineral nodules. These staining techniques were employed to monitor and assess the osteogenic differentiation of the rBMSCs over the course of the experiment.

### RNA-seq and data analysis

After 6 h of culturing with DMEM, PHA or FPHA extract, the macrophages were collected and total RNA was extracted as described before. The RNA sequencing was performed using the extracted total RNA by BGI Genomics, China. The thresholds for differentially expressed transcripts were set at *P* < 0.05 and logFC = 0.585. To gain insights into the biological functions and signaling pathways impacted by these differentially expressed transcripts, several analytical tools were employed. Gene Ontology (GO) and Kyoto Encyclopedia of Genes and Genomes (KEGG) enrichment analysis were performed, which provided information on gene functions and the involvement of specific pathways. Furthermore, Gene Set Enrichment Analysis (GSEA, v4.3.2), Cytoscape (v3.9.1), ClueGo (v2.5.9), and STRING (a database for protein-protein interactions, v11.5) were used to analyze the differences between the PHA and FPHA groups within pre-defined gene sets.

### Metabolism investigation of macrophages following culture with PHA or FPHA extract

#### Mitochondrial membrane potential (ΔΨm)

The ΔΨm of macrophages was assessed using the JC-1 Assay Kit (C2003S, Beyotime Biotechnology, China) following the manufacturer’s protocol. After a 6-hour and 1-day culture with PHA extract and FPHA extract, macrophages were rinsed twice with PBS and then incubated with the JC-1 staining working solution at 37 °C. Following a 20-minute incubation, the cells were rinsed twice with JC-1 dilution buffer and immediately observed using a fluorescent inverted microscope (Axio Vert.A1, Zeiss, Germany) for qualitative analysis of mitochondrial membrane potential. Additionally, flow cytometry (BD LSRFortessa, USA) was utilized for quantitative analysis of mitochondrial membrane potential.

#### Reactive oxygen species (ROS)

The intracellular ROS level was measured using the reactive oxygen species assay kit (C1300-1, Solelybio, China) following the manufacturer’s instructions. Macrophages were activated and cultured for 6 h and 1 day. Subsequently, the cells were rinsed twice with PBS and incubated with serum-free culture medium supplemented with 10 µM DCFH-DA probe. Following a 30-minute incubation at 37 °C in the dark, the cells were rinsed twice with PBS. For qualitative analysis, the cells were immediately observed using a fluorescent inverted microscope. For quantitative analysis, the cells were promptly collected for flow cytometry analysis. A positive control group involving direct H_2_O_2_ addition as a ROS source was omitted, as LPS stimulation effectively triggers ROS production [29].

#### Macrophage metabolism states

To assess the functional activity of macrophages in terms of oxidative phosphorylation and glycolysis, the real-time mitochondrial oxygen consumption rates (OCRs) and the real-time extracellular acidification rates (ECARs) were measured using the XF96 Seahorse analyzer (Agilent Technologies, USA) according to the manufacturer’s instructions. Macrophages were cultured in XF96 pro microplates at a density of 3.0 × 10^4^ cells per well. Following activation as previously described, the macrophages were cultured for 6 h and 1 day with DMEM, PHA extract, or FPHA extract.

To measure OCRs, the cell culture medium was replaced with Seahorse XF DMEM supplemented with 10 mM glucose, 1 mM pyruvate, and 2 mM glutamine. Subsequently, the following compounds were injected into their respective ports to achieve a final concentration of 1.5 µM oligomycin (port A), 1.0 µM carbonyl cyanide-4 (trifluoromethoxy) phenylhydrazone (FCCP, port B), and 0.5 µM rotenone and antimycin-A (port C).

For the assessment of ECARs, the cell culture medium was replaced with Seahorse XF DMEM containing 2 mM glutamine. Additionally, 10 mM glucose, 1.0 µM oligomycin and 50 mM 2-deoxy-D-glucose (2-DG) were added to each well.

### In vivo immunomodulatory behavior of FPHA

#### Animal surgery

In this study, male Sprague-Dawley rats aged 6 to 8 weeks were used to investigate the immune-modulatory process of FPHA. All animal surgical procedures were conducted following the approved protocols of the Institutional Animal Care and Use Committee of Sun Yat-sen University (approval number: IACUC-DB-16-0103). To create the critical size calvarial defect model, the rats were anesthetized and a 1.5 cm sagittal incision was made on the scalp. The calvarium was then exposed through blunt dissection. Using a 5 mm diameter trephine bur, two bilateral calvarial bone defects were created. The insertion of bone substitutes into the bilateral defects was done randomly according to allocation. After 7 days, all animals survived the procedure without any signs of diseases or complications. The grafts and surrounding tissue were carefully dissected. The harvested specimens were fixed by immersion in 4% paraformaldehyde in 0.1 M phosphate buffer (pH 7.2) for 24 h and were decalcified in a 10% EDTA solution for four weeks.

#### Immunohistochemical staining

For immunohistochemical staining, after antigen retrieval, the slides were blocked with a 5% BSA solution for 1 ​hour and incubated with mouse anti-CD68 (1:100, Invitrogen, USA), anti-iNOS (1:100, Abcam, UK), anti-CD163 (1:1000, Proteintech, USA), anti-TNFα (1:100, ZenBio, China), anti-IL1β (1:100, Proteintech, USA), anti-IL10 (1:100, Proteintech, USA), anti-TGFβ1 (1:100, Proteintech, USA), anti-MMP9 (1:100, Proteintech, USA), anti-OCN (1:100, Affinity, China), anti-HIF1α (1:100, Proteintech, USA) and anti-SDHB (1:100, Proteintech, USA) at 4 ​°C overnight. Goat anti-rabbit IgG (1:100, Servicebio, China) was used as a secondary antibody and the nuclei were stained using 2-(4-amidinophenyl)-6-indolecarbamidine dihydrochloride (DAPI, Servicebio, China). Images were captured using Aperio AT2 (Leica, German).

### Statistical analysis

In the study, all experiments were conducted at least three times to ensure reproducibility. The Shapiro-Wilk test was used to assess the normality of the data. If the data was found to be normally distributed, one-way analysis of variance (ANOVA) followed by the Bonferroni post hoc test was employed to determine statistical significance between more than two groups. The statistical analyses were conducted using GraphPad Prism software (version 9.0). The data are presented as the mean ± standard deviation (SD), and a *p*-value less than 0.05 was considered statistically significant.

## Result

### Characterization and biocompatibility of PHA and FPHA

**Fig. 1:**
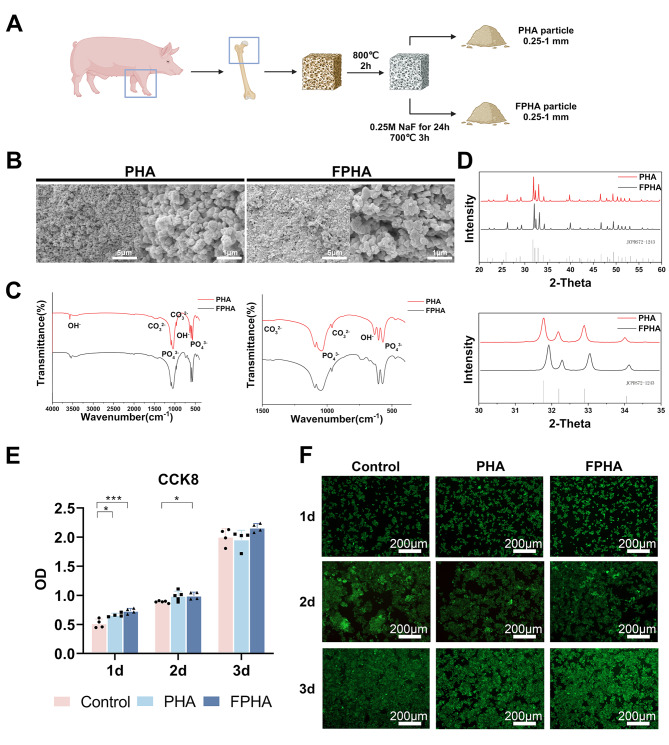
Characterization of fluorinated porcine hydroxyapatite. (**A**) The schematic diagram showed the workflow for the fabrication of FPHA. (**B**) SEM images displayed a spherical-shaped crystal of PHA (left) and a more rectangular crystal in FPHA (right). (**C**) FTIR spectra showed weakened intensity of the OH characteristic band at 3573 cm^− 1^ and 634 cm^− 1^ in FPHA. Instead, the band associated with OH-F or OH-F-HO became stronger at 3542 cm^− 1^ and 740 cm^− 1^, respectively. (**D**) XRD patterns demonstrated shifted reflection peaks towards higher diffraction angles after fluoridation. (**E**) Cell viability of macrophages cultured with PHA and FPHA extract for 1 to 3 days showed enhanced cell vitality and proliferation in the early stages of culture. (**F**) The live/dead staining assay showed a higher number of cells remained viable in the FPHA group

PHA and FPHA were synthesized using a chemical and thermal method (Fig. 1A). SEM analysis demonstrated a uniform distribution of crystals in both PHA and FPHA. PHA crystals displayed a spherical shape (left), whereas more rectangular crystals were observed in FPHA (right) (Fig. 1B). The EDS results indicated that the major chemical elements in PHA and FPHA were oxygen, calcium, carbon, and phosphorus, with sodium and magnesium detected at relatively low levels (Tables 1and Fig. S1).

**Table 1:** EDS results of PHA and FPHA

Material	Mol %						
	C	O	Na	Mg	*P*	Ca	Ca/*P*
PHA	13.94	60.75	0.29	0.54	9.44	14.73	1.56
FPHA	14.04	57.84	0.31	0.7	10.36	16.47	1.59

In the FTIR spectra, the vibrational bands corresponding to functional groups such as PO_4_^3−^ (1058 cm^− 1^ and 569 cm^− 1^), hydroxyls (OH, 631 cm^− 1^ and 3573 cm^− 1^), and CO_3_^2−^ (1415 cm^− 1^ and 1477 cm^− 1^) exhibited relatively consistent patterns for both PHA and FPHA. However, the intensity of the OH characteristic band at 3573 cm^− 1^ became weakened, while the band associated with OH-F or OH-F-HO became stronger at 3542 cm^− 1^ in FPHA. Another significant change was the disappearance of the absorption band around 634 cm^− 1^ attributed to OH, which was replaced by a new band around 740 cm^− 1^ after fluoridation (Fig. 1C).

The XRD patterns of both PHA and FPHA were consistent with the stoichiometric reference pattern of hydroxyapatite (JCPDS72-1243), indicating that both materials were crystallized in a pure phase. However, the reflection peaks shifted towards higher diffraction angles, indicating structural changes induced by the incorporation of fluoride (Fig. 1D).

In the cell culture experiments, the fluoride ion concentration in the medium was measured to be 0.19 ± 0.01 mg/L. In the CCK-8 assay, the cell viability of macrophages cultured with FPHA was significantly higher on day 1 and day 2 compared to the control group (Fig. 1E). This indicates that FPHA promote cell vitality and proliferation in the early stages of culture. The live/dead staining assay further confirmed the cytocompatibility of PHA and FPHA. During the 3-day culture period, the majority of cells exhibited green fluorescence. Additionally, the number of live cells was higher when cultured with the FPHA extract compared to the control and PHA (Fig. 1F). This suggests that FPHA may provide a more favorable microenvironment for cell survival and proliferation compared to PHA. This is an important prerequisite for a bone substitute, as its ability to support cell viability and proliferation is crucial for successful tissue integration and regeneration.

### FPHA promoted osteogenic differentiation via mediating macrophage polarization phenotype and conditioning an anti-inflammatory osteo-immune microenvironment

#### FPHA extract suppressed M1 polarization and induced macrophages M2 polarization

**Fig. 2:**
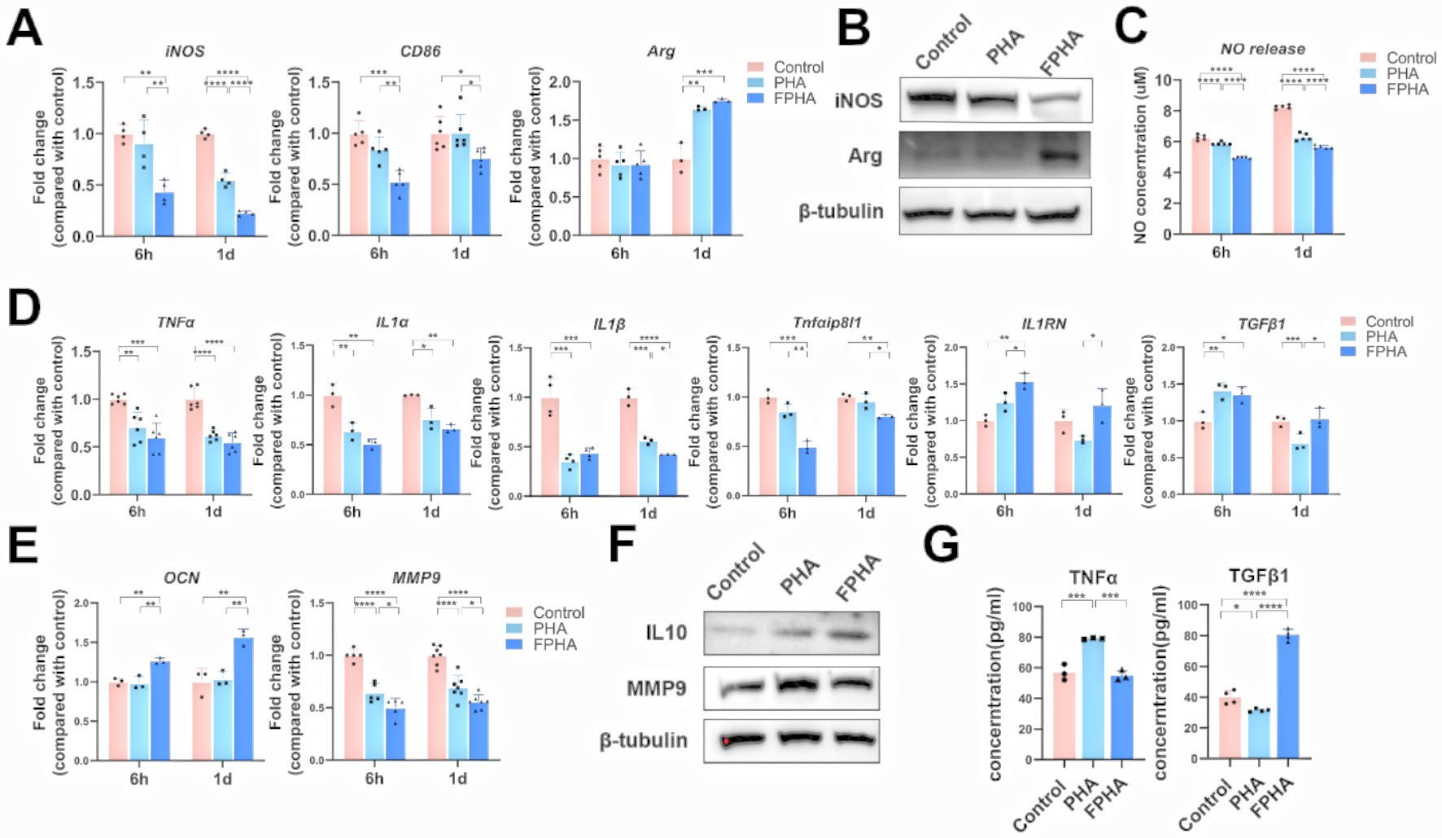
Macrophage response to FPHA. (**A**) The qRT-PCR analysis demonstrated that culturing with FPHA extract resulted in lower levels of M1 polarization-related genes (*iNOS* and *CD86*) and higher levels of M2 polarization-related gene (*Arg*). (**B**) Western blot analysis showed that cells cultured with FPHA extract exhibited inhibited expression of iNOS and enhanced expression of Arg. (**C**) NO release detection indicating lower levels of NO in cells cultured with FPHA extract. (**D**) The qRT-PCR analysis demonstrated inhibited expression of inflammatory genes and enhanced expression of anti-inflammatory genes in the FPHA group. (**E**) The qRT-PCR analysis presented downregulation of osteoclast-specific genes and upregulation of osteogenesis-related gene expression in the FPHA group. (**F**) Western blot analysis proved that cells cultured with FPHA extract exhibited inhibited expression of MMP9 and enhanced expression of IL10. (**G**) ELISA assay showed increased level of TGFβ1 and decreased level of TNFα in cells cultured with FPHA extract. (The data are shown as the mean ± SD (*n* ≥ 3); Statistical analysis was performed using ANOVA and multiple comparisons post-hoc tests (Tukey HSD). *p < 0.05, **p < 0.01, ***p < 0.001 and ****p < 0.0001)

The qRT-PCR analysis showed that culturing with FPHA extract significantly decreased the expression levels of M1 polarization-related genes, including *iNOS* and *CD86*, at 6 h and 1 day (Fig. 2A). On the other hand, the expression level of the M2 polarization-related gene arginase (*Arg*) increased after culturing with PHA or FPHA extract for 1 day, but no significant differences were found between the two groups (Fig. 2A).

Further western blot analysis demonstrated that the expression of iNOS was inhibited, while the expression of Arg was enhanced in macrophages cultured with FPHA extract compared to those cultured with PHA extract (Fig. 2B).

Additionally, a NO release detection assay revealed that a significantly lower level of NO was released after culturing with FPHA compared to PHA (Fig. 2C), which indicated M1 polarization suppression in macrophages [30].

These findings collectively confirmed that FPHA suppressed M1 polarization and induced macrophages toward M2 polarization, even with LPS pre-stimulation, further supporting the immunomodulatory effects of FPHA in promoting an anti-inflammatory macrophage polarization phenotype.

#### FPHA extract promoted a favorable osteo-immune microenvironment

Osteo-immune microenvironment plays a key role in bone regeneration [3]. The qRT-PCR analysis showed that both FPHA and PHA extract reduced the expression of inflammatory factors compared to control group, such as *TNFα, IL1α*, and *IL1β*. Culturing with FPHA extract resulted in lower expression levels of *Tnfαip8l1*, and inhibited expression of *IL1β* at 1 day in macrophages compared to PHA (Fig. 2D). Increased expression levels of anti-inflammatory genes, including *IL1RN* and *TGFβ1*, were observed in macrophages cultured with FPHA extract (Fig. 2D). Furthermore, the expression of the osteogenesis-related gene *OCN* was highly upregulated in cells cultured with FPHA extract, indicating enhanced osteogenesis. Conversely, the expression of the osteoclast-specific gene *MMP9* was relatively low (Fig. 2E).

Western blot analysis revealed that the anti-inflammatory factor IL10 was upregulated, while the osteoclast-specific factor MMP9 was downregulated in the FPHA group (Fig. 2F). For secretory proteins, a lower expression level of TNFα protein was detected in the supernatant of cell culture media after culturing with FPHA extract for 48 h (Fig. 2G). And the anti-inflammatory factor TGFβ1 was significantly upregulated in the FPHA group (Fig. 2G). It is worth noting that the expression of certain genes and proteins may not always perfectly correlate due to the involvement of multiple complex processes in the pathways from genes to proteins [31]. However, these findings collectively suggest that FPHA exhibits a enhanced osteoconductive and osteo-immunomodulatory capacity, promoting a favorable osteo-immune microenvironment. This implies that FPHA has the potential to enhance bone regeneration by regulating inflammation and facilitating osteogenesis.

#### FPHA extract promoted osteogenic differentiation via modulating macrophage polarization

**Fig. 3:**
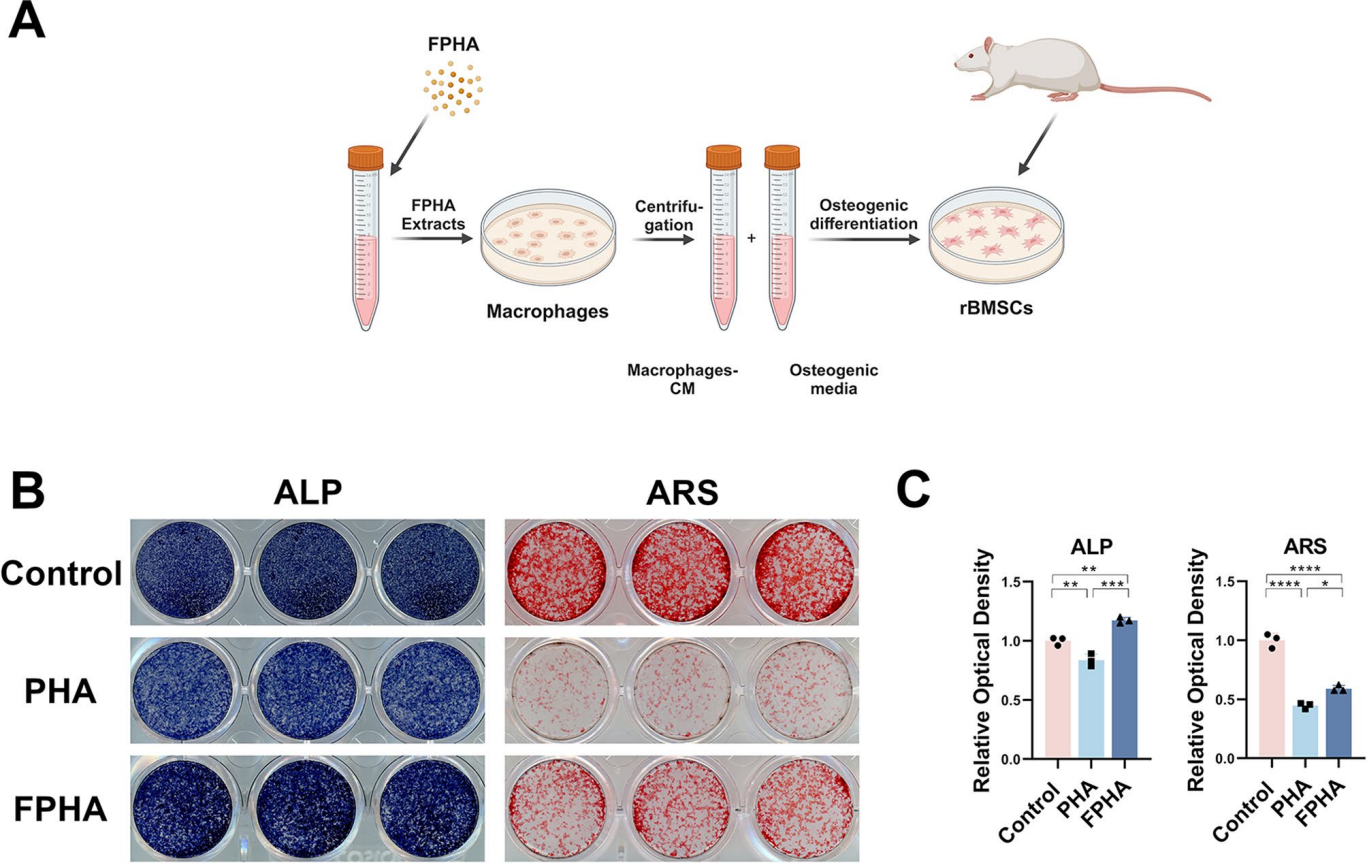
In vitro effects of FPHA on osteogenesis of rBMSCs. (**A**) Schematic representation of RAW264.7 macrophage conditioned medium extraction and preparation of osteogenic media for rBMSCs culture. (**B**) ALP and ARS staining showed a significant enhancement of osteogenic differentiation in the FPHA group. (**C**) Statistical analysis of ALP and ARS staining. (*p < 0.05, **p < 0.01, ***p < 0.001 and ****p < 0.0001)

To further elucidate the effect of FPHA on osteogenesis, we cultured rBMSCs in a co-stimulation medium (Fig. 3A). The results of ALP and ARS staining demonstrated a noticeable enhancement of osteogenic differentiation in the FPHA group (Fig. 3B).

## RNA-seq analysis indicated FPHA might mediate macrophage polarization through reprogramming cell metabolism

Based on the aforementioned findings, we can tentatively infer that FPHA suppressed M1 polarization and promoted M2 polarization of macrophages, thereby modulating the osteo-immune microenvironment and facilitating osteogenic differentiation. To further elucidate the underlying biological mechanisms, we conducted RNA sequencing analysis to investigate gene expression in macrophages cultured with FPHA extract.

**Fig. 4:**
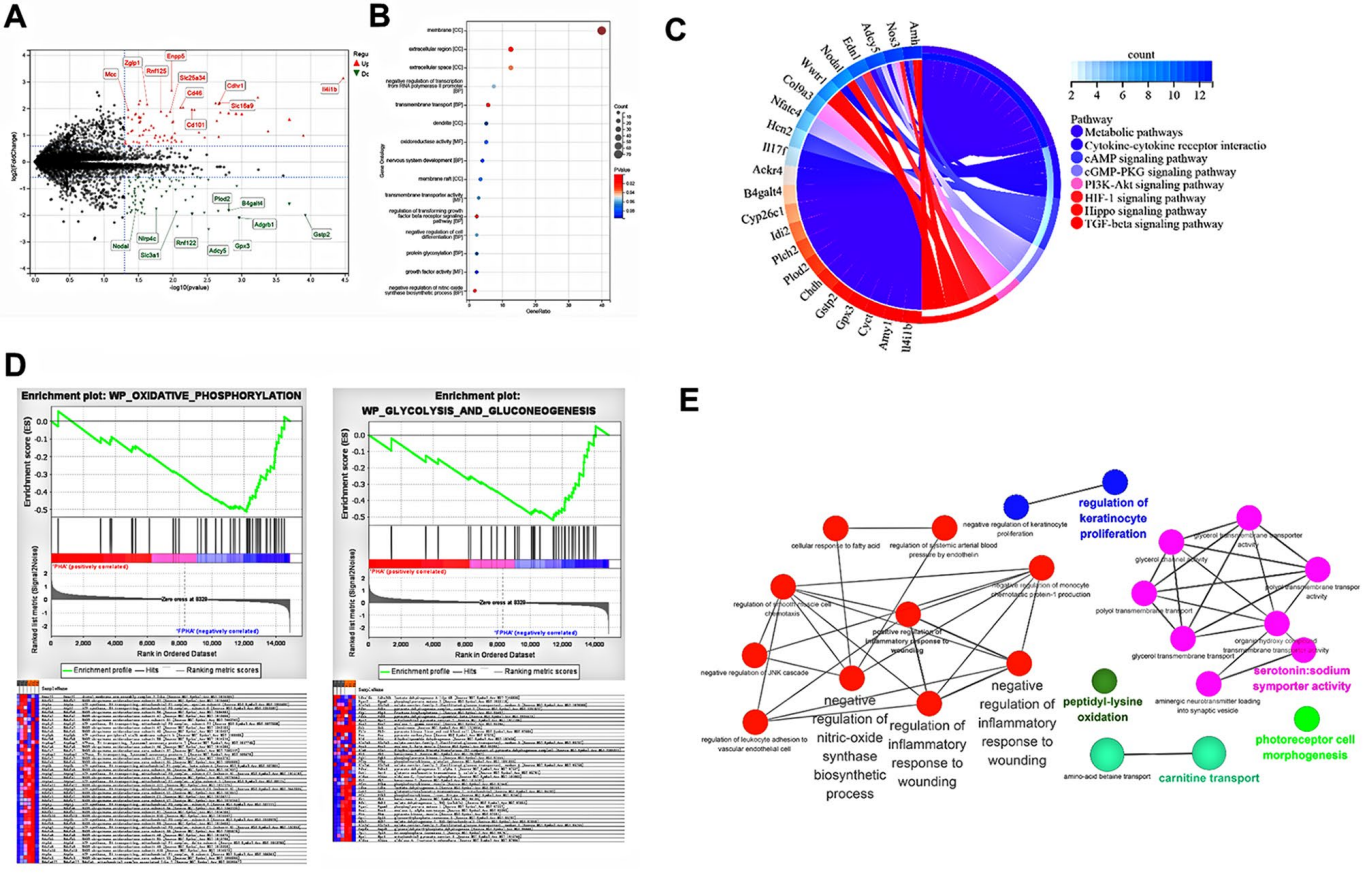
Culturing with FPHA extract enhanced OxPhos (associated with M2 Polarization) while attenuated glycolysis (associated with M1 Polarization) of macrophages, and downregulated the inflammatory response through RNA-seq analysis. (**A**) Volcano plot of RNA-seq analysis showed differentially expressed genes, with red representing upregulated genes and green representing downregulated genes in FPHA group. (**B**) GO enrichment analysis highlighted the enrichment of differentially expressed genes within the cellular component (CC), molecular function (MF), and biological processes (BP) categories. (**C**) The KEGG enrichment analysis demonstrated the potential active involvement of differentially expressed genes in the regulation of metabolic pathways. (**D**) GSEA presented that cells cultured with FPHA extract upregulated the oxidative phosphorylation pathway and the gluconeogenesis pathway. (**E**) Pathway enrichment analysis conducted using Cytoscape + ClueGo identified that FPHA downregulated the nitric-oxide synthase biosynthetic process and the inflammatory response to wounding

The differential gene expression analysis revealed distinct patterns, as depicted in the volcano plot (Fig. 4A). GO enrichment analysis highlighted the enrichment of differentially expressed genes primarily associated with the cell membrane, as observed in the cellular component category (Fig. 4B). At the molecular function level, these genes were associated with activities such as oxidoreductase activity, transmembrane transporter activity, and growth factor activity. In terms of biological processes, the enriched genes were involved in the regulation of TGFβ receptor signaling pathway and the negative regulation of nitric-oxide synthase biosynthetic process, corroborating our earlier observations regarding the macrophage response to FPHA. Moreover, the KEGG enrichment analysis suggested that these differentially expressed genes may play active roles in the regulation of metabolic pathways (Fig. 4C). To further investigate the specific metabolic pathways contributing to FPHA-mediated changes in macrophage polarization, we performed GSEA analysis. The results indicated upregulation of gene expression related to oxidative phosphorylation, and glycolysis and gluconeogenesis in the FPHA group (Fig. 4D), with the core enrichment genes suggesting the involvement of FPHA in ATP synthesis and gluconeogenesis. In general, gluconeogenesis is an anabolic pathway that reverses and does not occur simultaneously with the glycolytic pathway [32]. It is well-documented that macrophage polarization states are largely determined by mitochondrial functions and metabolic cascades [6, 12, 33]. M2 macrophages exhibit metabolic shift towards oxidative phosphorylation, while M1 macrophages rely on glycolysis and increased production of reactive oxygen species [12]. Hence, these findings suggest that FPHA may induce a metabolic shift from glycolysis to oxidative phosphorylation, facilitating the reprogramming of M1 macrophages into the M2 phenotype.

Moreover, pathway enrichment analysis conducted using Cytoscape + ClueGo identified that FPHA downregulated the nitric-oxide synthase biosynthetic process and the inflammatory response to wounding (Fig. 4E). Additionally, protein-protein interaction network analysis performed using STRING revealed that the associated proteins mainly localized to the cell membrane and may potentially influence macrophage polarization through nitric oxide synthesis (Fig. S2).

### FPHA remodeled macrophage metabolism from glycolysis to OxPhos

#### FPHA extract strengthen macrophage mitochondrial function

**Fig. 5:**
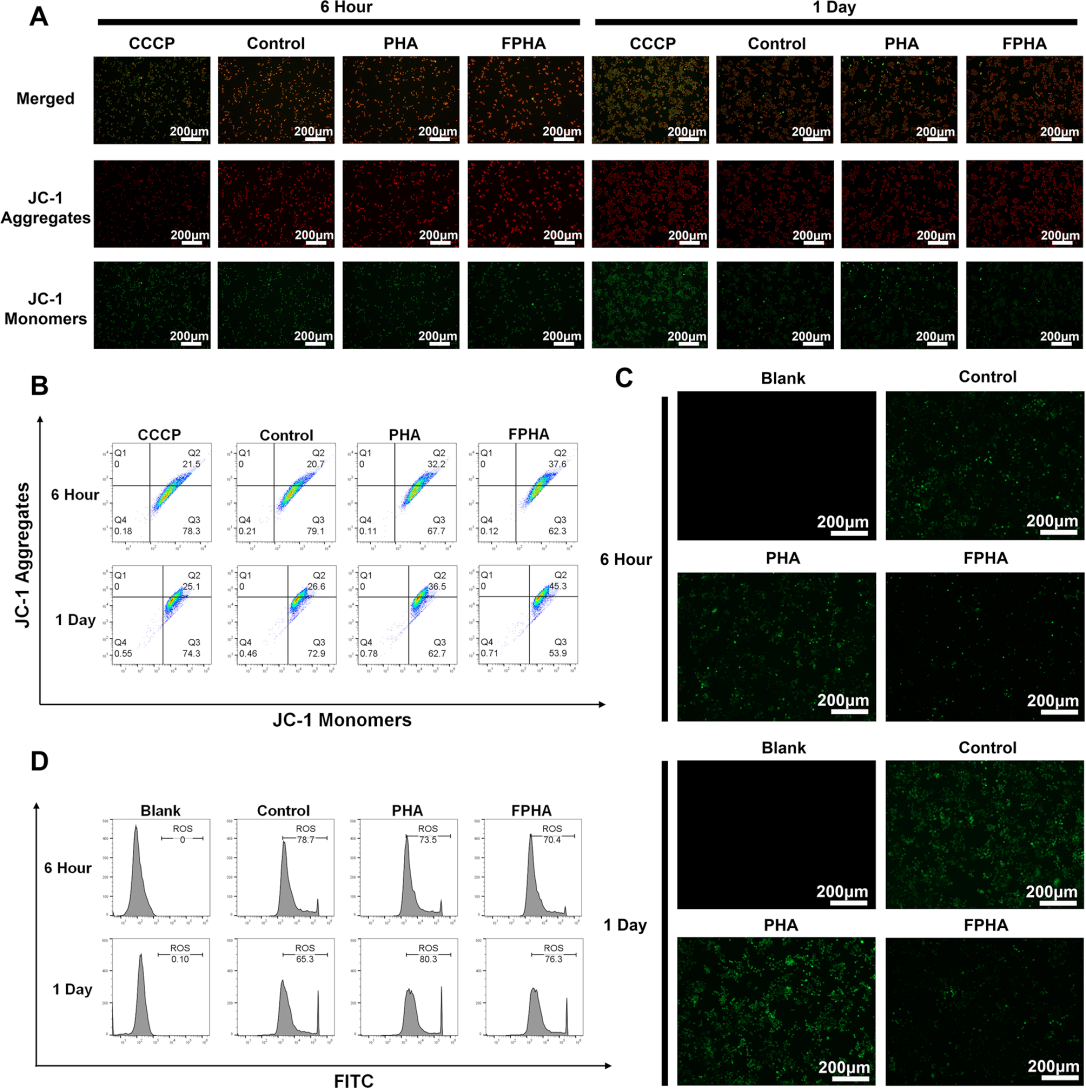
Enhanced mitochondrial function in cells cultured with FPHA extract. (**A**) Evaluation of mitochondrial membrane potential using JC-1 probe revealed increased formation of JC-1 aggregates in cells treated with FPHA extract, leading to higher intensity of red fluorescence and lower intensity of green fluorescence (JC-1 monomers). (**B**) Flow cytometry analysis further supported the enhancement effect of FPHA on mitochondrial energy synthesis. (**C**) The FPHA-treated group exhibited a significant decrease in the percentage of DCF-labeled cells (green fluorescence) compared to both the control and PHA groups. (**D**) Flow cytometry analysis further confirmed the inhibitory effect of FPHA on ROS production

A decrease in ΔΨm is considered a characteristic of mitochondrial dysfunction and early apoptosis, leading to reduced energy synthesis [34]. To assess ΔΨm in macrophages, JC-1 staining was employed. Cells with high ΔΨm display red fluorescence due to the formation of JC-1 aggregates, while cells with low ΔΨm exhibit green fluorescence as JC-1 remains in its monomeric form. In validation experiments conducted at both 6 h and 1 day, cells treated with FPHA extract exhibited higher intensity of red fluorescence and lower intensity of green fluorescence, particularly at the 1-day time point (Fig. 5A). This observation was further confirmed by flow cytometry analysis (Fig. 5B).

Mitochondria are a major source of the ROS, and it is worth noting that ΔΨm plays a crucial role in governing ROS production [35]. To evaluate intracellular ROS levels, we utilized the DCFH-DA probe. The FPHA group displayed a significant decrease in the percentage of DCF-labeled cells (green fluorescence) compared to both control and PHA groups (Fig. 5C), indicating a smaller amount of ROS. Further flow cytometry analysis also confirmed that FPHA effectively reduced intracellular ROS levels (Fig. 5D).

The aforementioned data collectively suggest that FPHA extract can enhance ΔΨm and reduce ROS production in macrophages. Thus, FPHA demonstrates a robust ability to strengthen macrophage mitochondrial function.

#### FPHA extract enhanced OxPhos and suppressed glycolysis

Different metabolic strategies can redirect macrophages polarization, for example, increasing oxidative phosphorylation allow M2 reprogramming [12]. Previous RNA-seq results indicated that FPHA upregulated OxPhos pathways and downregulated glycolysis pathways.

**Fig. 6:**
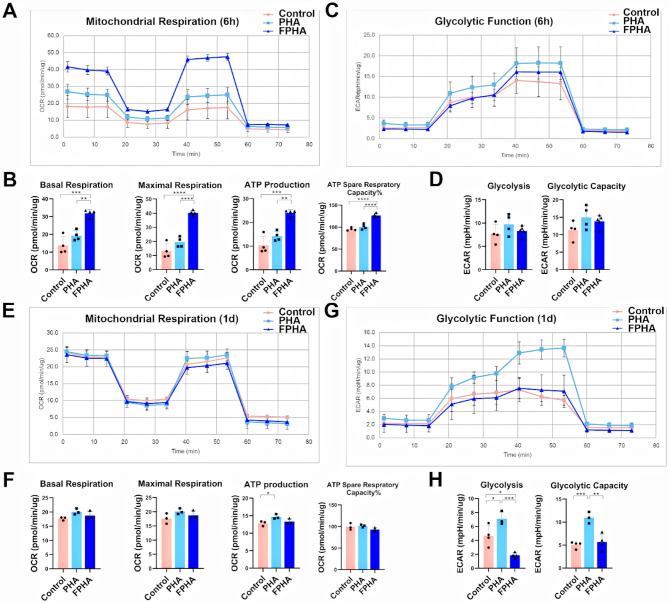
Enhanced OxPhos and suppressed glycolysis in cells cultured with FPHA extract. (**A**) Real-time OCARs of macrophages after 6 h of culturing, in response to the sequential injection of Oligo, FCCP, and rotenone (cell mitochondrial stress test). (**B**) Quantification of basal respiration, maximal respiration, ATP production and ATP spare respiratory capacity in macrophage mitochondria after 6 h of culturing. The FPHA group exhibited significantly higher levels of basal respiration, maximal respiration, ATP production, and ATP spare respiratory capacity compared to the control and PHA groups. (**C**) Real-time ECARs of macrophages after 6 h of culturing, in response to the sequential injection of glucose, Oligo and 2-DG (glycolysis stress test). (**D**) Quantification of glycolysis and glycolytic capacity after 6 h of culturing. (**E**) Real-time OCARs and (**F**) real-time ECARs of macrophages after 1 day of culturing. (**G**) Quantification analysis of OxPhos and (**H**) glycolysis parameters for cells cultured for 1 day. The FPHA group exhibited significantly higher levels of basal respiration, maximal respiration, ATP production, and ATP spare respiratory capacity compared to the control and PHA groups. (The data are shown as the mean ± SD (*n* ≥ 3); Statistical analysis was performed using ANOVA and multiple comparisons post-hoc tests (Tukey HSD). *p < 0.05, **p < 0.01, ***p < 0.001 and ****p < 0.0001)

To validate these effects, we first recorded the real-time OCRs of macrophages treated with FPHA extract after sequential treatment with oligomycin, FCCP, rotenone, and antimycin A. These compounds allowed the analysis of OxPhos parameters in the macrophages. After a 6-hour culture with FPHA extract (Fig. 6A), the macrophages exhibited significantly higher levels of basal respiration, maximal respiration, ATP production, and ATP spare respiratory capacity% (Fig. 6B). However, the OxPhos levels were similar among the three groups after 1 day of culturing (Fig. 6C and D).

In parallel, we also measured the real-time ECARs in response to sequential injection of glucose, oligomycin, and 2-DG to analyze the glycolytic parameters of the macrophages. Although glycolysis was similar at 6 h among the different groups (Fig. 6E and F), macrophages cultured with FPHA extract showed significantly lower glycolysis and glycolytic capacity after 1 day of culturing (Fig. 6G and H).

### FPHA fostered a favorable osteo-immune microenvironment in rat critical size calvarial defects

**Fig. 7:**
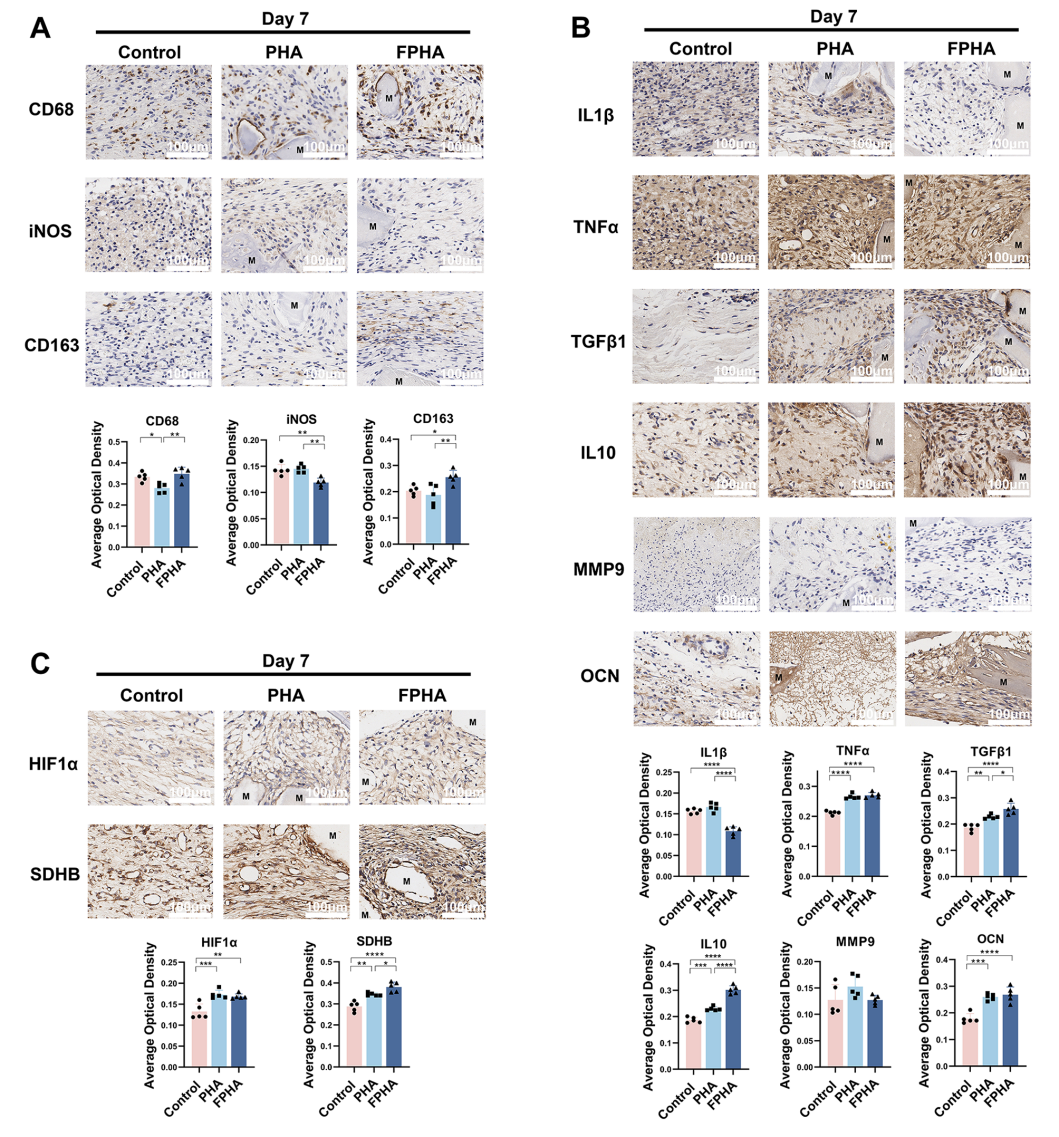
Fluorinated porcine hydroxyapatite (FPHA) fostered a favorable osteo-immune microenvironment in rat critical size calvarial defects. (**A**) Immunohistochemistry analysis demonstrated the presence of CD68^+^, iNOS^+^ and CD163^+^ macrophages on day 7. (**B**) Immunohistochemistry analysis revealed the presence of IL1β^+^, TNFα^+^, TGFβ1^+^, IL10^+^, MMP9^+^ and OCN^+^ cells on day 7. (**C**) Immunohistochemistry analysis showed the presence of HIF-1α^+^ and SDHB^+^ cells on day 7. (The data are shown as the mean ± SD (*n* ≥ 3); Statistical analysis was performed using ANOVA and multiple comparisons post-hoc tests (Tukey HSD). *p < 0.05, **p < 0.01, ***p < 0.001 and ****p < 0.0001)

Firstly, we investigated the response of macrophages to FPHA. Immunohistochemical staining results indicated that the expression level of the macrophage marker CD68 was higher in the FPHA group (Fig. 7A), compared to PHA. In addition, FPHA was found to significantly reduce the expression of the M1 polarization marker iNOS and enhance the expression of the M2 polarization marker CD163 compared to PHA (Fig. 7A), suggesting that FPHA had a greater ability to recruit macrophages and promote their polarization towards the M2 phenotype.

Additionally, we investigated the impact of FPHA on macrophage polarization and its subsequent effects on osteo-immunomodulation. The expression levels of the inflammation marker IL1β were notably decreased in the FPHA group, while both the FPHA and PHA groups showed an increase in expression levels of TNFα. Additionally, the FPHA group demonstrated increased expression of anti-inflammatory markers, including TGFβ1 and IL10 (Fig. 7B). However, no significant differences were observed between the PHA and FPHA groups regarding the expression of the osteoclast-specific factor MMP9 and the osteogenesis-related factor OCN (Fig. 7B). Furthermore, based on the aforementioned RNA-seq analysis, we assessed the expression of HIF1α and SDHB. It was observed that HIF1α was upregulated in both PHA and FPHA groups (Fig. 7C), and the expression of SDHB was significantly enhanced in the FPHA group (Fig. 7C).

## Discussion

The development of smart biomaterials as bone substitutes for regeneration has garnered significant attention in recent years due to their osteo-immunomodulatory properties. Macrophage polarization plays a fundamental role in the quality and effectiveness of injury repair [5]. Fluoride exhibits diverse effects on immune cell metabolism and function [14, 19, 20] and has been proposed as a potential regulator of bone tissue regeneration. Thus, the modification of biomaterials with fluoride may be a promising strategy for modulating the osteoimmunology environment by regulating immunometabolism. In our previous studies [21, 23–27], FPHA was synthesized and demonstrated enhanced physicochemical and biological properties. In the present study, we synthesized the optimum concentration of FPHA accordingly [24], and evaluated its physicochemical properties to confirm the incorporation of fluoride ions. Additionally, we explored the potential underlying mechanisms contributing to these improvements.

Fluoride plays a crucial role in the regulation of bone tissue regeneration and exerts its effects in a concentration-dependent manner within the human body [18]. The fluoride ion concentration in the medium was measured to be 0.19 ± 0.01 mg/L in the above cell culture experiment, which falls within the range of fluoride ion concentrations demonstrated to promote macrophage M2 polarization based on previous study [36]. Moreover, it has been demonstrated that FPHA has the ability to continuously release fluoride ions [25]. These findings suggested that FPHA has the potential to modulate macrophage polarization through the release of fluoride ions, providing a continuous immunomodulatory effect.

The aforementioned findings from the present study suggested that FPHA had the ability to suppress the M1 polarization of macrophages while promoting their polarization toward the M2 phenotype, leading to the creation of a favorable osteo-immune microenvironment. Bone remodeling is intricately and spatiotemporally regulated by the balance between osteoclast-mediated resorption and osteoblast-mediated formation [37]. Therefore, we cultured rBMSCs in a co-stimulation medium to evaluated the effect of FPHA on osteogenesis. Importantly, it should be noted that PHA showed inhibitory effects on osteogenic differentiation as observed through ALP and ARS staining. Additionally, cells cultured with PHA extract exhibited higher expression levels of TNFα and MMP9 proteins, while the expression of TGFβ1 was suppressed. These observations suggested that although PHA, as a bone substitute intended to enhance bone regeneration, seemed to induce a heightened inflammatory reaction and had a detrimental impact on bone remodeling at the early stage. Referring to the previous studies [38, 39], it is postulated that the inflammatory response elicited by PHA may result from the release of small calcium phosphate (CaP) particles that are not efficiently filtered by a 0.22 μm filter. Importantly, CaP particles, particularly amorphous calcium phosphate (ACP) nanoparticles, have been shown to activate inflammatory cells and induce the release of inflammatory mediators [38]. Moreover, it has been demonstrated that the inflammatory response to CaP particles is influenced by their physicochemical properties [40]. The exciting result of our study was the significant attenuation of the inflammatory effects triggered by CaP particles through the application of FPHA, ultimately promoting a favorable osteo-immune microenvironment and facilitating osteogenic differentiation.

The RNA-seq results suggested that the aforementioned changes in osteo-immune microenvironment may be related to cellular metabolism. Mitochondria, often referred to as the “powerhouse of the cell,” are essential organelles involved in OxPhos and are a primary source of ROS [41]. Changes in cellular metabolism can influence mitochondrial functions [42]. The effect of fluoride ions is time dependent [19, 22]. To further explore the metabolic changes, we conducted measurements at both 6 h and 1 day to obtain a more comprehensive understanding of the temporal effects of FPHA extract on macrophage mitochondrial function. Based on the mitochondrial metabolism states, it can be concluded that FPHA may have a temporal effect on the metabolic shift of macrophages. After a 6-hour culturing, FPHA promoted OxPhos and related parameters. However, the effects on OxPhos were no longer significant after 1 day. Additionally, FPHA reduced glycolysis and the glycolytic capacity of macrophages after 1 day. The ratio of oxidative phosphorylation to glycolysis was found to be upregulated at both 6 h and 1 day, indicating increased tendency towards M2 polarization in macrophages.

In our previous studies, we have already showcased the superior efficacy of FPHA over PHA in enhancing bone repair within critical size calvarial defects in SD rats [21, 26] at 6 weeks and 12 weeks. Additionally, FPHA promoted bone regeneration in the mandible defects in Beagle dogs, with significant improvements observed at 12 weeks [27]. Building upon these findings, we carried out in vivo experiments using SD rat calvarial defects to further validate the observed outcomes from in vitro experiments in the present study. Based on the mechanism of bone healing, the period between days 5–10 during bone defect repair is when metabolism, inflammation, and the aggregation of osteogenesis-related cells converge [43, 44]. The implanted bone substitutes are infiltrated by migrating mesenchymal stem cells, which undergo differentiation over a course of approximately 7 days into chondroblasts and chondrocytes, and subsequently into osteoblasts and osteoclasts, leading to the gradual resorption of cartilage and deposition of new bone [45]. Therefore, we detected the surrounding tissues on day 7 to capture this critical stage.

The changes in macrophage response and osteo-immune microenvironment in vivo were consistent with those in the in vitro experiments. Therefore, we further investigated the alterations in cellular metabolism. In the aforementioned RNA-seq analysis, KEGG enrichment analysis revealed that differentially expressed genes were associated with the HIF-1 signaling pathway. Previous studies have demonstrated that elevated levels of ROS activated hypoxia-inducible factor 1 alpha (HIF1α), a pivotal transcription factor involved in the regulation of proinflammatory gene expression [46, 47]. Thus, we assessed the expression of HIF1α and it was observed that both PHA and FPHA groups exhibited upregulation of HIF1α. Hypoxia-inducible factors (HIFs) are recognized as principal regulators in the response to hypoxic conditions, with particular emphasis on the role of HIF1α [48]. Given the compromised blood supply, the provisional callus initially experiences a hypoxic microenvironment [49]. Despite the potential of HIF1α to induce inflammation during the initial inflammatory phase, it can also adapt to the hypoxic conditions by facilitating VEGF production, thereby promoting callus angiogenesis [49]. In the control group, the defect was in an aerobic environment with soft tissue containing blood supply through the vessels originating from the surrounding tissue. However, in both the PHA and FPHA groups, the bone defects were filled with bone substitutes, resulting in insufficient blood supply. Consequently, the upregulation of HIF1α facilitated vascular invasion and supported bone regeneration. It is worth noting that although HIF1a expression was upregulated, its downstream inflammatory factor IL1β [50] was significantly downregulated both in vitro and in vivo in the FPHA group. This finding suggested that FPHA did not induce an increase in inflammatory levels.

OxPhos is a highly efficient energy-generating process involving electron flow through five main respiratory chain complexes (complexes I, II, III, IV, and V) located in the inner mitochondrial membrane [46, 51]. Among these complexes, succinate dehydrogenase (SDH) is unique as it participates in both the TCA cycle and the electron transport chain (ETC) [33, 52, 53], both of which are crucial for OxPhos. SDH catalyzes the conversion of succinate to fumarate in the TCA cycle, while also facilitating electron transfer, thus promoting respiratory chain activity [46, 53]. SDHB is a secondary electron transfer subunit that tightly binds to SDHA and interacts with SDHC and SDHD to form a stable subunit complex [54, 55]. Additionally, succinate, an inflammatory metabolite, accumulates during macrophage activation and affects HIF1α activity [50] and ROS levels [53]. To validate the in vivo impact of FPHA on mitochondrial function, we performed immunohistochemical staining analysis on SDHB expression in the FPHA group. The results demonstrated upregulation of SDHB, confirming that FPHA may enhance OxPhos through SDHB.

Taken together, the present study supported the notion that FPHA might upregulate SDHB. This promotion, on one hand, enhanced the TCA cycle and the electron transport chain, thereby facilitating OxPhos and suppressing the expression of ROS. On the other hand, the oxidation of succinic acid by SDHB reduced intracellular succinate accumulation, leading to reduced cellular inflammation. Consequently, FPHA contributed to the polarization of macrophages towards the M2 phenotype and created a favorable osteogenic microenvironment.

Inflammation is a universal response. Regardless of the location of bone defects, bone tissue regeneration progresses through three continuing and overlapping phases: inflammation, regeneration and remodeling [56]. Although appropriate inflammation is vital for bone defect healing and essential for normal tissue repair, heightened inflammatory state hinder the pro-regenerative environment and thereby impede bone tissue repair [57]. In clinical practice, excessive bone defects, local inflammation such as periodontitis, and systemic disease such as diabetes, may contribute to further elevated inflammation levels during bone defect repair. In such instances, macrophages are more inclined to M1 polarization, leading to a surge in glycolysis and a suppression of OxPhos. Of the various materials available for bone defect repair in medical therapy, biological-derived hydroxyapatite is the most commonly used material because their composition mimics the mineral bone phase [58]. However, the currently clinically used materials primarily act as scaffolds, with relatively insufficient osteo-immune regulatory properties. At this time, FPHA exhibits a certain targeted therapeutic potential. Through its metabolic-immunoregulatory properties, FPHA prompts surrounding macrophages to shift from glycolysis to OxPhos, timely eliminating the pro-inflammatory stage following the acute inflammation. This not only prevents the progression of acute inflammation, triggered by the implantation of bone replacement materials, into chronic inflammation, but also fosters a regenerative environment conducive to bone formation.

Currently, there are many other modification methods available to enhance the osteo-immune regulatory properties of materials, such as piezoelectric stimulation [15], addition of stem cells [59] and green chemical method [60]. Among these, nanobiomaterials, especially green nanomaterials have attracted considerable attention [61, 62]. The previous study [63] has indicated that PLGA/nanofluorohydroxyapatite can better promote cell viability and enhance mechanical properties. It is widely recognized that the retention of carbonate in biological-derived hydroxyapatite is crucial for maintaining its biological and physicochemical properties [64]. The low-temperature green synthesis approach favors the retention of carbonate [65] and provides valuable insights for further improving FPHA.

Our research also has some limitations. Due to the particulate nature of the bone substitute material, it was challenging to maintain stable cell culture conditions [66]. Therefore, we utilized cell culture with the extract of the material following the ISO/EN 10993-5 standard [67]. Despite its limitations, this approach is widely accepted for assessing particulate bone substitutes due to its ability to mimic the effects of materials in the in vivo environment, following the release of soluble components [26, 68, 69]. This allows for the assessment of the modulation of the osteo-immune microenvironment by a variety of trace element ions released by the materials.

Additionally, the immunohistochemical results obtained in vivo did not entirely correspond with the findings from our in vitro experiments. The in vivo TNFα expression level was increased in all material groups. This may be due to the fact that the implantation of materials triggers acute inflammation, leading to an upregulation of TNFα expression. However, FPHA suppresses inflammation by upregulating SDHB and related metabolic pathways, which is primarily mediated through the regulation of IL1β expression levels [6, 13], rather than TNFα. Both in vitro and in vivo experiments have indicated a downregulation of IL1β expression, with a more pronounced decrease observed in vivo. Therefore, this downregulation ensures that the anti-inflammatory effects of FPHA remain unaffected. Meanwhile, the in vivo expression level of MMP9 was not inhibited, and there was no statistical difference among the three groups. These discrepancies may be attributed to the immune response elicited by the bone substitute material as a foreign body in vivo, which may not solely involve macrophages. However, overall results still suggest that FPHA can influence macrophage polarization through the regulation of metabolic shift.

In light of the aforementioned limitations, further investigation is needed to delve deeper into the molecular mechanisms through which FPHA affects SDHB expression and induces its immunomodulatory effects. Different models could be used to explore efficacy of FPHA in various pathological conditions, such as periodontitis. Additionally, preliminary radiographic results of the ongoing clinical trials indicate that the effectiveness of FPHA in the repair of alveolar bone defects is comparable to that of bovine bone materials commonly used in clinical practice. However, the long-term clinical bone maintenance necessitates continuous observation and evaluate.

## Conclusion

In summary, our study revealed that FPHA induced a metabolic shift in macrophages from glycolysis to OxPhos. This metabolic shift led to the suppression of ROS levels, potentially mediated by upregulated SDHB expression. As a result, FPHA lead to significant suppression of M1 macrophage polarization and promotion of M2 macrophage polarization. This resulted in the creation of an anti-inflammatory and osteogenic microenvironment, facilitating rBMSCs osteogenic differentiation. The findings of this study offer valuable insights into the impact of incorporating an optimal concentration of fluoride on immunometabolism and macrophage mitochondrial function. These results have important implications for the development of fluoride-modified bone regenerative biomaterials that leverage immunometabolism as a mechanism for enhanced bone regeneration.

## Electronic supplementary material

Below is the link to the electronic supplementary material.

Supplementary Material 1

## Data Availability

The data presented in the current study will be available from the corresponding author on reasonable request.

## Abbreviations

FPHA: Fluorinated porcine hydroxyapatite
ROS: Reactive oxygen species
PPP: Pentose phosphate pathway
OxPhos: Oxidative phosphorylation
TCA: Tricarboxylic acid
NO: Nitric oxide
rBMSCs: Rat bone marrow-derived mesenchymal stem cells
ALP: Alkaline phosphatase
ARS: Alizarin red S
ΔΨm: Mitochondrial membrane potential
OCRs: Oxygen consumption rates
ECARs: Extracellular acidification rates
FCCP: Carbonyl cyanide-4 (trifluoromethoxy) phenylhydrazone
2-DG: 2-deoxy-D-glucose
CaP: Calcium phosphate
ACP: Amorphous calcium phosphate
SDH: Succinate dehydrogenase
ETC: Electron transport chain
