# Correction: Fluorinated hydroxyapatite conditions a favorable osteo-immune microenvironment via triggering metabolic shift from glycolysis to oxidative phosphorylation

**DOI:** 10.1186/s12967-025-06386-6

**Published:** 2025-04-11

**Authors:** Kaidi Chen, Seongmin Ha, Leyao Xu, Chengwu Liu, Yuanxiang Liu, Xiayi Wu, Zhipeng Li, Shiyu Wu, Bo Yang, Zhuofan Chen

**Affiliations:** 1 Hospital of Stomatology, Sun Yat-Sen University, Guangzhou, China; 2 Guangdong Provincial Key Laboratory of Stomatology, Guangzhou, China; 3 Guanghua School of Stomatology, Sun Yat-Sen University, Guangzhou, China

**Correction: Journal of Translational Medicine (2024) 22:437** 10.1186/s12967-024-05261-0

Following publication of the original article [1], we have been notified that Fig. 5 was published incorrectly.

It is now as follows:

**Fig. 5:**
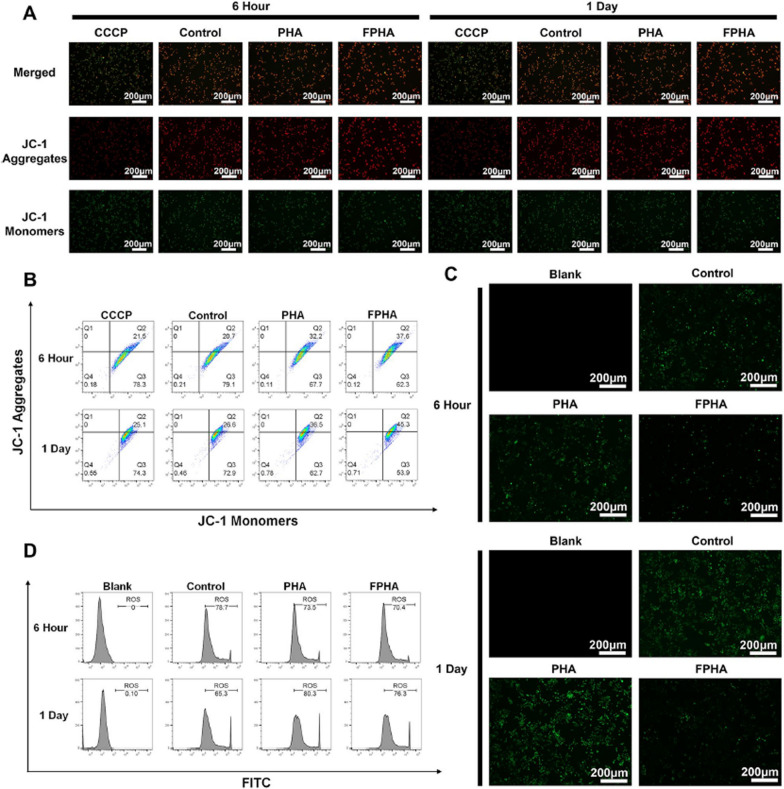
Enhanced mitochondrial function in cells cultured with FPHA extract. **A** Evaluation of mitochondrial membrane potential using JC-1 probe revealed increased formation of JC-1 aggregates in cells treated with FPHA extract, leading to higher intensity of red fluorescence and lower intensity of green fluorescence (JC-1 monomers). **B** Flow cytometry analysis further supported the enhancement effect of FPHA on mitochondrial energy synthesis. **C** The FPHA-treated group exhibited a significant decrease in the percentage of DCF-labeled cells (green fluorescence) compared to both the control and PHA groups. **D** Flow cytometry analysis further confirmed the inhibitory effect of FPHA on ROS production

Figure 5 should be as follows:

**Fig. 5:**
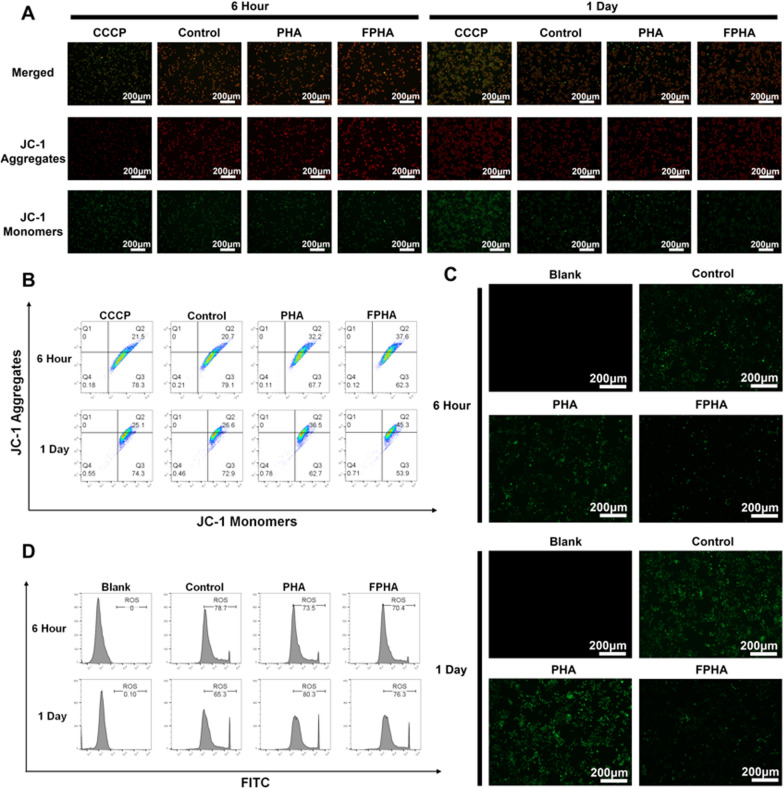
Enhanced mitochondrial function in cells cultured with FPHA extract. **A** Evaluation of mitochondrial membrane potential using JC-1 probe revealed increased formation of JC-1 aggregates in cells treated with FPHA extract, leading to higher intensity of red fluorescence and lower intensity of green fluorescence (JC-1 monomers). **B** Flow cytometry analysis further supported the enhancement effect of FPHA on mitochondrial energy synthesis. **C** The FPHA-treated group exhibited a significant decrease in the percentage of DCF-labeled cells (green fluorescence) compared to both the control and PHA groups. **D** Flow cytometry analysis further confirmed the inhibitory effect of FPHA on ROS production

The original article was updated.
