# Supplementary material for: Fluorinated hydroxyapatite conditions a favorable osteo-immune microenvironment via triggering metabolic shift from glycolysis to oxidative phosphorylation
Source: J Transl Med. 2024 May 8;22:437. doi: 10.1186/s12967-024-05261-0 (PMC11077739; doi:10.1186/s12967-024-05261-0)
Supplement: Supplementary file 1 — Supplementary Material 1 [file 12967_2024_5261_MOESM1_ESM.docx]

# Supplementary Material

| **Table S1.** Primer used in the qRT-PCR assays | |
| --- | --- |
| Gene | Primer sequences |
| ***iNOS*** | F: 5’-CAGAAGTGCAAAGTCTCAGACAT-3’  R: 5’-CAGGAGCGTTAGTGACAGCAG-3’ |
| ***CD86*** | F: 5’-CTGCTCATCATTGTATGTCAC-3’  R: 5’-ACTGCCTTCACTCTGCATTTG-3’ |
| ***Arg*** | F: 5’-ACAAGACAGGGCTCCTTTCAG-3’  R: 5’-TGAGTTCCGAAGCAAGCCAA-3’ |
| ***TNFα*** | F: 5’-CTGAACTTCGGGGTGATCGG-3’  R: 5’-GGCTTGTCACTCGAATTTTGAGA-3’ |
| ***IL1α*** | F: 5’-CGAAGACTACAGTTCTGCCATT-3’  R: 5’-GACGTTTCAGAGGTTCTCAGAG-3’ |
| ***IL1β*** | F: 5’-TGGAGAGTGTGGATCCCAAG-3’  R: 5’-GGTGCTGATGTACCAGTTGG-3’ |
| ***TNFαip8l1*** | F: 5’-GTTTGTGGACAATACCAGCAGT-3’  R: 5’-GTTCTTCACTACCCTCTGTGC-3’ |
| ***IL6*** | F: 5’-ATAGTCCTTCCTACCCCAATTTCC-3’  R: 5’-GATGAATTGGATGGTCTTGGTCC-3’ |
| ***TGFβ1*** | F: 5’-CAGTACAGCAAGGTCCTTGC-3’  R: 5’-ACGTAGTAGACGATGGGCAG-3’ |
| ***IL1RN*** | F: 5’-GCTCATTGCTGGGTACTTACAA-3’  R: 5’-CCAGACTTGGCACAAGACAGG-3’ |
| ***MMP9*** | F: 5’-TTCGACTTGAAGTCTCAGAAGGTG-3’  R: 5’-TTCGACTTGAAGTCTCAGAAGGTG-3’ |
| ***OCN*** | F: 5’-CTGACAAAGCCTTCATGTCCAA-3’  R: 5’-GCGCCGGAGTCTGTTCACTA-3’ |
| ***GADPH*** | F: 5’-TCAGCAATGCCTCCTGCAC-3’  R: 5’-TCTGGGTGGCAGTGATGGC-3’ |





## **Figure S1.** EDS results indicated that the major chemical elements in PHA and FPHA are similar.


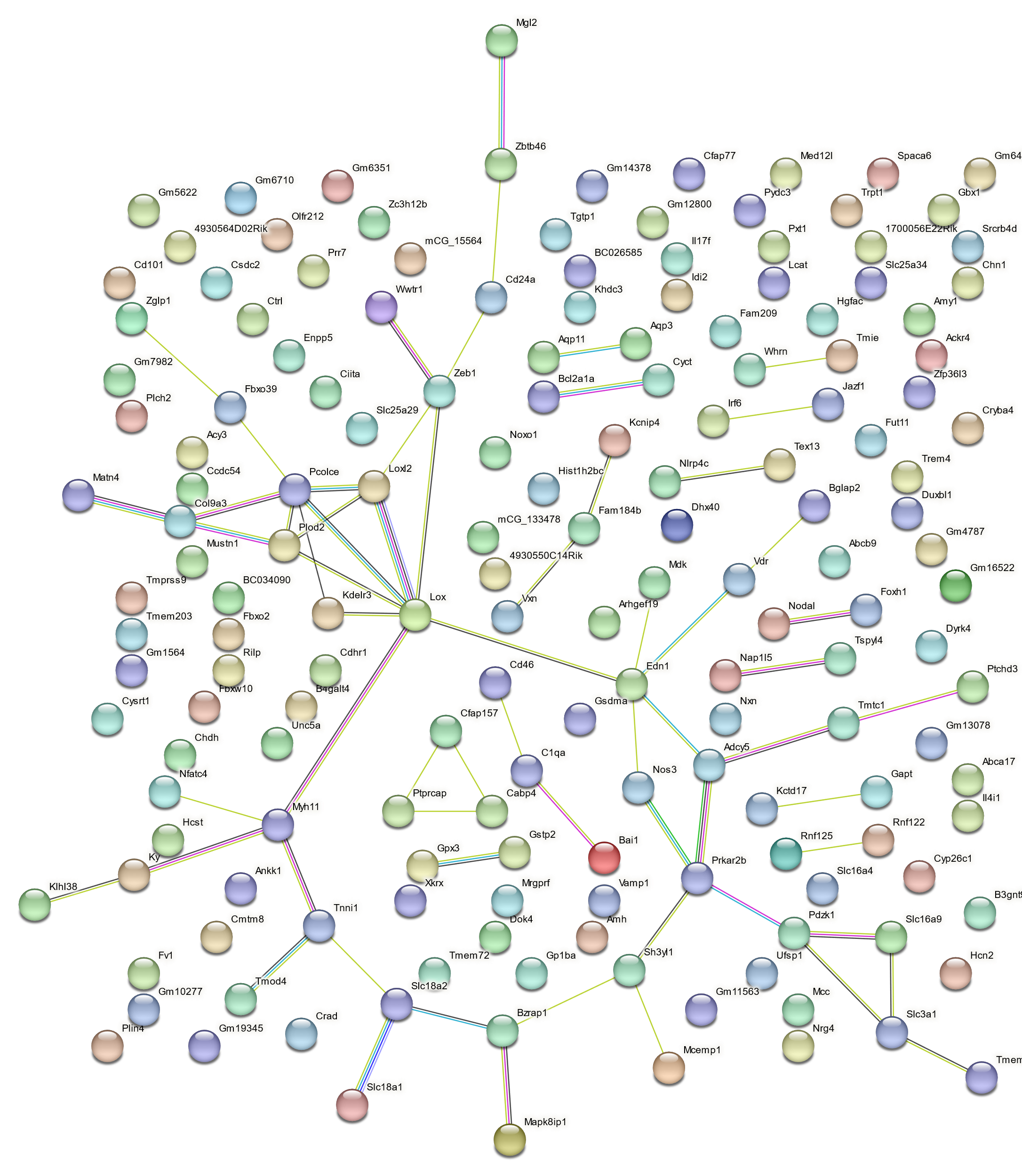


## **Figure S2.** STRING results revealed the protein-protein interaction network.
